# Male involvement in female partners’ screening for breast and cervical cancers in Southwest Nigeria

**DOI:** 10.1371/journal.pone.0284141

**Published:** 2023-05-10

**Authors:** Ifeoma Peace Okafor, Folayemi Oyinkansola Kukoyi, Oluchi Joan Kanma-Okafor, Michael Orji Izuka

**Affiliations:** Department of Community Health & Primary Care, College of Medicine, University of Lagos, Mushin, Lagos, Nigeria; Debre Tabor University, ETHIOPIA

## Abstract

**Background:**

Breast and cervical cancers are in the top 10 most common cancers in women globally and the most common cancers in Nigerian women. The incidences have been rising steadily over the years. Involvement of men as key players in reproductive health issues has been receiving global attention especially in low and middle-income countries.

**Aim:**

To assess male involvement in their female partners’ screening for breast and cervical cancers in Southwest, Nigeria.

**Method:**

This was a community-based, cross-sectional study that employed a multi-stage sampling method to select 254 men who were married or in steady relationships in Lagos State, Southwest Nigeria. Data were collected from June to October 2018 using a semi-structured interviewer-administered questionnaire, analyzed using Epi Info version 3.5.1 and summarized with mean and standard deviation. Chi-square test was used for bivariate statistics, and the p-value of ≤0.05 was considered statistically significant. Multivariable logistic regression was used for predictor variables of male involvement in screening.

**Results:**

29.5% of the respondents had good knowledge of breast and cervical cancers and screening and majority (85.5%) had a positive attitude towards screening. Only few, 19.3% and 15.7% had provided money for breast and cervical cancer screening respectively. Most men, 75% and 87.4% respectively had not accompanied their wife/female partner for breast and cervical cancer screening, while almost half (49.2%) and one-third (33.5%) respectively, had encouraged their female partners to screen for breast and cervical cancers. Overall, only about half, 138 (54.3%) of the men were considered ‘involved’ in their female partners’ screening for breast and cervical cancers. Male involvement was significantly associated with screening for female cancers (χ2 = 77.62, p = 0.001). Older age group (AOR = 2.64, 95% CI: 1.3–4.9), higher educational attainment (AOR = 3.51, 95% CI: 1.14–10.73), and positive attitude (AOR = 2.48, 95% CI:1.16–5.33) were found to be the predictors of male involvement.

**Conclusion:**

Community-based programs for males, especially the younger and less educated, should be implemented to increase their involvement. It is also suggested that mass media messages be spread and online platforms be explored in order to increase men’s awareness and participation in female cancer screening.

## Introduction

Cancer is among the top three causes of premature mortality (i.e. between the ages of 30–69), accounting for 1 in 7 premature deaths overall and 1 in 4 fatalities from noncommunicable diseases [1, 2]. Worldwide breast cancer is the most common cancer overall and the most common cancer in women with 2.3 million new cases in 2020 [3, 4]. It is estimated to be responsible for 15.5% of all cancer deaths among women [4]. Cervical cancer is the seventh most common cancer overall and the fourth most common cancer in women with hundreds of thousands of cases and deaths [3, 4]. Breast cancer and cervical cancer are the two most frequent cancers affecting women in SSA including Nigeria and the incidences and mortality are predicted to rise [3, 4]. High morbidity and mortality could be reduced greatly using preventive health methods such as HPV testing, pap test and mammography screening, the goal of which is to detect cell changes early enough for successful treatment before cancer develops [5, 6].

Men are increasingly being encouraged to promote access to reproductive health (RH) services [6, 7]. Calls were made decades ago to involve men as key stakeholders in RH issues because they are major decision makers at the household and family levels [8, 9]. Ensuring equity in the access to sexual and RH services was also highlighted in the Sustainable Development Goals (SDGs) 3 and 5 [10]. Men’s roles in their partners’ RH experiences range from joint decision-making or granting permission to the provision of financial support and transportation [11]. Recent reviews show that lack of knowledge about female cancers and the role of screening, unsupportive partners and families, cost and fear are major barriers to screening for breast and cervical cancers among women in LMICs [12, 13]. The need for spousal approval is also a barrier to cervical cancer screening among Nigerian women [14]. The World Health Organization (WHO) has since recommended the involvement of men in the prevention of breast and cervical cancers in low and middle-income countries (LMICs) as with other aspects of women’s RH [6]. For screening, their involvement may be in the form of financial provision, accompaniment, or encouragement of their wives or female partners [15–17]. Regardless of these global efforts, many men do not have adequate knowledge of RH issues including breast and cervical cancers, thus limiting their involvement in screening [16–20]. Male involvement is seen as a multi-pronged concept in the literature, but a multi-dimensional evidence-based set of indicators is lacking, making its measurement difficult. Generally, it has frequently been measured by actions like physical presence at health services, financial support or providing transportation. Most studies also did not have men themselves as study respondents [21].

In view of the fore-going, this study aimed to assess male involvement in their female partners’ screening for breast and cervical cancers using men as study participants. We also assessed their knowledge about female cancers and attitudes towards screening as important factors which may influence their involvement. Findings will enable the design or scaling of targeted interventions to increase spousal involvement in screening to achieve the much-desired reduction in the incidence and prevalence of breast and cervical cancers in LMICs.

## Study design & methods

Lagos State is Nigeria’s economic and commercial capital. The State is located in the South–Western part of Nigeria, on the narrow plain of the Bight of Benin. Though Lagos State is the smallest state in Nigeria it has the highest urban population, which is 27.4% of the national estimate. Kosofe is one of the 20 Local Government Areas in Lagos State. Based on the 2006 National Population census, Kosofe housed 682,772 people (358,935 males and 323,837 females). However, the population is projected to have risen to up to 1 million people based on estimated annual population growth rate of 2.5%. There are many government and private owned health facilities which offer breast and cervical screening services in the area.

The study was a community-based, cross-sectional study carried out among men in Kosofe Local Government Area to assess their involvement in female partners’ screening for breast and cervical cancers. Only men who were married or in a steady relationship and residing in the study area were included in the study. Men who were unable to respond to questions appropriately or were temporary residents/visitors to the study area were excluded.

Sample size was determined using Cochran’s formula (n = z^2^p(1-p)/e^2^) [22]. The calculation was based on: prevalence of good knowledge (p) of 20% obtained from a similar study [23], standard normal deviate (z) at 95% confidence (1.96) and 5% margin of error (e) resulting in a minimum sample size of 246. To compensate for possible non-responses and incomplete questionnaires, 10% was added, making a total of 271 administered on the field. Eventually, 254 were filled satisfactorily and analyzed yielding a response rate of 93.7%.

A multistage sampling technique was used to select respondents in this order: First stage involved selection of wards, then secondly, selection of streets, followed by third stage, selection of house with numbered houses on each street as sampling frame. Last was selection of respondents (fourth stage). At each stage, the simple random sampling method was used except for the selection of houses where systematic sampling was employed to select every kth house following a calculated sampling interval—total number of houses on the street is the numerator with the required number of houses on that street is the denominator. The interval varied according to the total number of houses on the street.

Data were collected by face-to-face interviews using a semi-structured interviewer-administered questionnaire developed based on adaptations from previous studies done in Ghana, Kenya, and Malawi [20, 23, 24], and inclusion of other variables (S1 File). The questionnaire was divided into sections to gather information on respondents’ socio-demographic and economic characteristics, knowledge of breast and cervical cancers and screening, attitude towards screening for these cancers, and involvement in female partners’ health generally, and screening for female cancers. Knowledge was assessed with a series of questions on the symptoms, cause/risk factors, availability of treatment, and purpose and benefits of screening for breast and cervical cancers. Attitude was assessed with statements such as ‘I believe that screening for breast cancer without first observing symptoms is a waste of time’, ‘Screening for cervical cancer is unnecessary and a waste of money’,’ ‘It is not necessary for me to accompany my female partner to screen for breast cancer’, ‘I will never allow a male health professional screen my female partner for breast and cervical cancers’, ‘My wife/partner does not need to obtain my approval before screening for breast and cervical cancers’. Respondents’ lifelong involvement in their wife’s or partner’s screening for female cancers was assessed with ‘Yes’ or ‘No’ responses to questions like: ‘Have you provided financially for your wife/partner to be screened for cervical cancer?’, ‘Have you ever sacrificed your time to accompany your wife/partner to be screened for breast cancer?’, Have you ever encouraged your wife/partner to screen for cervical cancer?’. The questionnaire was assessed for content validity by a team of experts in the College of Medicine, University of Lagos and then pretested among a similar population in another setting outside the study area.

Data were analyzed using Epi Info version 3.5.1 and SPSS software. Descriptive statistics such as percentage and mean were used to summarize the data. The chi-square test was used to test the association between selected variables with the level of significance set at 5% (p<0.05).

Ethical approval (No ADM/DST/HREC/APP/204) for the study was obtained from the Lagos University Teaching Hospital Health Research Ethics committee (HREC) before commencement of the study. Written consent was obtained from each respondent before the commencement of the interview. The respondents were assured of the highest level of confidentiality on information received through anonymity as names, phone numbers, and addresses were not required during the study. Interviews were conducted privately.

### Measurements

The respondents’ knowledge of breast and cervical cancers was assessed with a set of knowledge questions which were scored and graded to measure their level of knowledge. A score of 1 was allocated for each correct answer and 0 for wrong answers. A total knowledge score of 32 (100%) was the maximum score attainable. Good knowledge was determined if the respondent answered 50% or more (score of 16–32) of the questions correctly, and poor knowledge was determined if the respondent answered less than 50% (score of <16) correctly [25, 26].

The men’s attitude to female cancer screening was assessed by their level of agreement to a series of statements on a three-point Likert scale. Responses to the negative Likert statements were scored as follows: Strongly agree- 1, Slightly agree- 2, Don’t agree at all- 3. The reverse was used to score positive statements. A maximum total attitude score of 36 and a minimum score of 12 with a midpoint attitude score of 24. Grading was achieved as follows:

Positive attitude: scores ≥ to the median score of—27
Negative attitude: scores < the median score of—27

Male involvement in this context was defined as involvement by a male spouse/partner in at least any one of the following: encouragement to screen, financial support for screening, and accompaniment to cancer screening services. A score of 1 was allocated for each affirmative response to any of these and 0 for negative response. A total score of zero was categorized as ‘not involved’ while a total score of at least 1 was categorized as ‘involved.’

### Outcome variables

Primary outcome: The proportion of respondents involved in female partners’ screening for breast and cervical cancers.
Secondary outcomes: The proportion of respondents with good knowledge of breast and cervical cancers, and the proportion of respondents with a positive attitude towards breast and cervical cancer screening.

## Results

### Socio-demography

A total of 254 questionnaires were administered and all were properly filled and analyzed. Almost half (46.9%) of the respondents are between the ages of 31–40. The mean age was 40.27 ± 8.41 standard deviation. Most (88.6%) of the men are married or cohabiting had been in a marriage/relationship for 1–10 years (69.3%). The majority (94.1%) had a post-secondary education. The predominant religion was Christianity (89.0%). Only 5.1% of the men were unemployed compared to the 94.9% who had employment. Almost 30% of the men had an estimated monthly income in the range of N201,000 –N500,000. The median estimated monthly income was 200,000 Naira and interquartile range (IQR) 100,000–400,000 (Table 1).

**Table 1:** Sociodemographic characteristics.

Variables	Frequency (N = 254)	%
**Age group (years)**		
21–30	21	8.3
31–40	119	46.9
41–50	83	32.7
51–70	31	12.1
Mean age = 40.27 ± 8.41 SD		
**Marital status**		
Single	21	8.3
Married/co-habiting	225	88.6
Separated/divorced/widowed	8	3.1
**Duration of marriage/relationship (years)**		
1–10	176	69.3
11–20	57	22.4
21–30	14	5.5
31–40	7	2.8
**Level of education**		
Primary education	2	0.8
Secondary education	13	5.1
Post-secondary education	239	94.1
**Ethnicity**		
Yoruba	180	70.9
Igbo	44	17.3
Hausa	2	0.8
Others	28	11.0
**Religion**		
Christian	226	89.0
Muslim	28	11.0
**Employment status**		
Employed	241	94.9
Unemployed	13	5.1
**Average Monthly income (Naira)**		
<100,000	58	22.8
N100,000–500000	144	56.7
>500,000	37	20.5
**Median income (IQR)**	200,000 (100,000–400,000)	
**Number of wives/partners**		
1	247	97.2
2	6	2.4
3	1	0.4
**No of children**		
0	46	18.1
1	44	17.3
2	78	30.7
3	49	19.3
>4	37	14.6
Total	254	100.0
**Characteristics of spouse**		
**Age (years)**		
<30	69	27.2
31–40	124	48.8
41–50	45	17.7
>51	16	6.3
**Highest level of education**		
No-formal	1	0.4
Primary school	3	1.2
Secondary school	15	5.9
Post-secondary education	235	92.5
**Employment status**		
Employed	205	80.7
Unemployed	49	19.3

Almost all (97.2%) of the men are in monogamous relationships. About one in three 30.7% of them had 2 children while 14.6% had 4 or more children. Almost half (48.8%) of the partners of the participants were in the age range of 31–40 years. The majority (92.5%) of the partners had post-secondary education and were employed (80.7%). The majority (89.0%) of the men had gone for check-ups in a health facility before, but only 17.3% had ever gone for any form of cancer screening (Table 1).

### Knowledge of breast and cervical cancers

Results of men’s knowledge of common female cancers are shown in Tables 2 and 3. Almost all (98.8%) had heard of breast cancer and the first source of information was mass media (35.0%). The majority (82.7%) knew it as a disease in which cells in the breast grow out of control. The most mentioned symptoms were a lump in the breast (82.3%) and breast/nipple pain (37.4%), while 18.5% didn’t know any symptoms. The most mentioned risk factors for breast cancer were family history (48.8%) and genetics (44.9%). Slightly above one-fifth (22.4%) do not believe breast cancer could be treated. Most (94.5%) of the respondents had heard of breast cancer screening, and 85.0% of those who had heard of it, knew the purpose is for the detection of lumps/tumors. The majority (88.6%) knew that regular screening has benefits (Table 2).

**Table 2:** Knowledge of breast cancers.

Variables	Frequency	(%)
**Ever heard of breast cancer**	251	98.8
**First source of information (n = 251)**		
Health professional	72	28.3
Friends/family	44	17.3
Mass media	89	35.0
Survivors	8	3.1
Internet	36	14.2
Others	2	0.8
**Meaning of breast cancer**		
A disease in which cells in the breast grow out of control	210	82.7
* **Symptoms of breast cancer**		
Change in size or shape of breast	76	29.9
Dimpling of breast	43	16.9
Nipple discharge	77	30.3
Change in areola	16	6.3
Breast or nipple pain	95	37.4
Lump in breast	209	82.3
Others	23	9.1
I don’t know	12	4.7
* **Risk factors for breast cancer**		
Family history of breast cancer	124	48.8
Taking oral contraceptives	46	18.1
Aging	36	14.2
Smoking and alcohol consumption	81	31.9
Genes	114	44.9
Being overweight	13	5.1
Early menarche	11	4.3
Lack of physical activity	27	10.6
Others	28	11.0
I don’t know	47	18.5
**Breast cancer can be treated**	194	76.4
**Ever heard of breast cancer screening**	240	94.5
**Purpose of breast cancer screening (n = 240)**		
Detection of lumps/tumours	216	90.0
**Regular screening has benefits**	225	88.6

*Multiple response

**Table 3:** Knowledge of cervical cancer.

Variables	Frequency	(%)	95% CI
**Ever heard of cervical cancer**	218	85.8	
**First source of information on cervical cancer (n = 218)**			
Health professional	69	31.2	
Friends/family	32	14.6	
Mass media	80	36.7	
Survivors	3	1.3	
Internet	31	14.2	
Others	4	1.8	
**Meaning of cervical cancer**			
Abnormal growth of cells in the cervix	168	66.1	
**Cause of cervical cancer**			
Too much sex	6	2.4	
HPV	94	37.0	
Poor personal hygiene	9	3.5	
Abortion	12	4.7	
I don’t know	80	31.5	
Others	18	7.1	
* **Symptoms of cervical cancer**			
Pelvic pain	96	37.8	
Bleeding after intercourse	39	15.4	
Bleeding after period	41	16.1	
Increased unusual vaginal discharge	58	22.8	
Pain during sex	51	20.0	
I don’t know	66	26.0	
Others	17	6.7	
* **Risk factors for cervical cancer**			
HPV	98	38.6	
Multiple sex partners	76	29.9	
HIV	25	9.8	
I don’t know	77	30.3	
Others	22	8.7	
**Cervical cancer can be treated**	156	71.2	
**Ever heard of cervical cancer screening**	188	85.8	
**Purpose of cervical cancer screening (n = 188)**			
Detection of abnormal changes to the cervix/early detection of precancerous cells	155	82.4	
I don’t know	33	17.5	
Others	1	0.5	
**Overall knowledge of breast and cervical cancers**			
Good	75	29.5	23.9–35.5
Poor	189	70.5	64.5–76.1

*Multiple responses

Table 3 shows that 85.8% of the men had heard of cervical cancer, and the first source of information was mass media (31.5%). Two-thirds (66.1%) knew cervical cancer to be abnormal growth of cells in the cervix. Only 37.0% knew the cause of cervical cancer to be HPV. The most mentioned symptom was pelvic pain (37.8%), while 26.0% didn’t know any symptoms of cervical cancer. The most common risk factors mentioned were HPV (38.6%) and having multiple sex partners (29.9%). The majority (71.2%) of the men knew cervical cancer could be treated. Most of the men had heard of cervical cancer screening (85.8%), and 82.0% of those who had heard of it knew the purpose as ‘detection of abnormal changes to the cervix or early detection of precancerous cells’. Only 29.5% (N = 75) of the respondents had good knowledge on breast and cervical cancers and screening.

### Men’s attitude to female cancer screening

Most men disagreed with the statement that screening for breast (79.5%) and cervical (79.1%) cancers without observing symptoms first was a waste of time. Most agreed that screening for breast (96.5%) and cervical (95.3%) cancers were necessary. About half disagreed with the suggestion that it was not necessary to accompany their partners to screen for breast (55.5%) and cervical (54.3%) cancers. Most men (68.9%) disagreed with the statement ‘I should not interfere with my partner’s screening for breast and cervical cancer’. Most men (60.6%) will not object to a male health professional screening their partner for breast and cervical cancers. As much as a quarter (25.6%) of the men expects that their partner must obtain approval from them before the screening. Overall, the majority (79.5%, n = 202) of the men had a positive attitude towards breast and cervical cancer screening (Table 4).

**Table 4:** Men’s attitude to female cancer screening.

Statements	Agree(%)	Slightly agree (%)	Don’t agree at all (%)
Screening for breast cancer without first observing symptoms is a waste of time	30 (11.8)	22 (8.7)	202 (79.5)
Screening for cervical cancer without observing symptoms is a waste of time	28 (11.0)	25 (9.8)	201 (79.1)
Screening for breast cancer is necessary	245 (96.5)	7 (2.8)	2 (0.8)
Screening for cervical cancer is necessary	242 (95.3)	9 (3.5)	3 (1.2)
I don’t need to accompany my wife/partner to screen for breast cancer	47 (18.5)	66 (26.0)	141 (55.5)
I don’t need to accompany my wife/partner to screen for cervical cancer	49 (19.3)	67 (26.4)	138 (54.3)
I should not interfere with my wife/partner’s screening for breast and cervical cancers	56 (22.0)	23 (9.1)	175 (68.9)
I will not wait for long while accompanying my wife/partner to screen for breast and cervical cancers	54 (21.3)	48 (18.9)	152 (59.8)
I will never allow a male health professional to screen my female partner for breast and cervical cancers	27 (10.6)	73 (28.7)	154 (60.6)
My wife/partner does not need to obtain my approval before screening for breast and cervical cancers	146 (57.5)	43 (16.9)	65 (25.6)
I will leave my wife/partner if she is ever diagnosed with breast cancer	3 (1.2)	9 (3.5)	242 (95.3)
I will leave my wife/partner if she is ever diagnosed with cervical cancer	7 (2.8)	8 (3.1)	239 (94.1)
**Overall attitude**	**Frequency**	**Percentage**	**95% CI**
Positive	216	85	80.1–89.2
Negative	38	15	10.8–19.9

### Male involvement in breast and cervical cancer screening

Table 5 contains information on male involvement in their wife’s/female partner’s health in general and screening for female cancers. Most of the men (85.8%) said screening for breast and cervical cancers was the decision of both partners. Only few, 19.3% and 15.7% had provided money for breast and cervical cancer screening respectively. Most men, 75% and 87.4% respectively had not accompanied their wife/female partner for breast and cervical cancer screening, while almost half (49.2%) and one-third (33.5%) respectively, had encouraged their female partners to screen for breast and cervical cancers. Overall, only about half, 138 (54.3%) of the men were considered involved in their female partners’ screening for breast and cervical cancers ie playing at least one role in financial provision, accompaniment, and encouragement.

**Table 5:** Male involvement in female partners’ health and cancer screening.

Male involvement in female partners’ health			
Variables	Frequency	%	95% CI
Ever accompanied your partner to a health care facility	227	89.4	
Ever stayed home to care for your partner when she was ill	216	85.0	
Ever provided financially for your partner to visit a health specialist	242	95.3	
**Male involvement in female partners’ screening**			
**Variables**			
**Screening is the decision of:**			
Woman alone	28	11.0	
Man alone	8	3.1	
Both partners	218	85.8	
Provided money for my wife/partner to screen for breast cancer	49	19.3	
Provided money for my partner to screen for cervical cancer	40	15.7	
Accompanied my wife/partner to screen for breast cancer	38	15.0	
Accompanied my wife/partner to screen for cervical cancer	32	12.6	
Encouraged my wife/partner to screen for breast cancer	125	49.2	
Encouraged my partner to screen for cervical cancer	85	33.5	
**Overall involvement in female cancer screening**			
Yes	138	54.3	47.9–60.6
No	116	45.7	39.4–52.1
**My partner has been screened for breast cancer**			
Yes	106	41.7	
No	101	39.8	
I don’t know	47	18.5	
**Breast cancer screening result (n = 106)**			
Positive	3	2.8	
Negative	102	96.2	
I don’t know	1	0.9	
**My partner has been screened for cervical cancer**			
Yes	58	22.8	
No	134	52.8	
I don’t know	62	24.4	
**Cervical cancer screening result (n = 58)**			
Negative	58	100.0	

Only 41.7% of the respondents said their partner had been screened for breast cancer, and 22.8% for cervical cancer.

### Association of male involvement with screening

As shown in Table 6, financial assistance of the man had a significant positive association with female screening for cancer (χ2 = 92.54, p = 0.001). Same significant association was observed for accompaniment to the health facility (χ2 = 68.56, p = 0.001) and encouragement to screen (χ2 = 56.19, p = 0.001). Overall, male involvement significantly influenced screening (χ2 = 77.62, p = 0.001).

**Table 6:** Association between male involvement and screening for female cancers.

	Screened for Cancer			
	Yes	No	χ2	p-value
**Financial assistance**			92.54	0.001^†^
Yes	56(100.0)	0(0.0)		
No	55(27.8)	143(72.2)		
**Accompanied to health facility**			68.56	0.001^†^
Yes	44(100.0)	0(0.0)		
No	67(31.9)	143(68.1)		
**Encouraged Screening**			56.19	0.001
Yes	86(66.7)	43(33.3)		
No	25(20.0)	100(80.0)		
**Overall male involvement**			77.62	
Involved	95(68.8)	43(31.2)		0.001
Uninvolved	16(13.8)	100(86.2)		

^†^ Fisher’s exact p-value

### Predictors of male involvement in screening

Older age (male) predicted male involvement in female partners’ screening for cancer (AOR 3.4 CI 1.67–6.92). Men who attained post-secondary education had nearly 5 times higher odds of involvement in their partners’ screening and this relationship was statistically significant (AOR 4.72 CI 1.32–16.85) Having a positive attitude to female partner’s cancer screening equally had a significant positive relationship with involvement in screening (AOR 2.48 CI 1.16–5.33) (Table 7).

**Table 7:** Predictors of male involvement in female cancer screening.

	Male involvement					
	Yes (%)	No(%)	χ2	P	AOR(95% C1)	p
**Age group (years)**						
≤45	97(49.2)	100(50.8)			1	
>45	41(70.1)	16(29.9)	9.17	0.002	3.40(1.67–6.92)	0.001
**Educational level**						
≤Secondary	4(26.6)	11(73.4)			1	
Post Secondary	134(56.1)	105(43.9)	4.97	0.026	4.72(1.32–16.85)	0.017
**Ethnicity**						
Yoruba	109(60.6)	71(39.4)			2.63(1.47–4.71)	0.001
Others	29(39.2)	45(60.8)	9.64	0.019	1	
**Attitude**						
Positive	124(57.4)	92(42.6)	5.50	0.019	2.48(1.16–5.33)	0.020
Negative	14(36.8)	24(63.2)			1	

## Discussion

Men’s involvement in their female partners’ screening for breast and cervical cancers was inadequate. The young age of the respondents reflects the demographic profile of Nigeria which depicts a young population. Most of our respondents had heard of breast cancer and their first source of information was mass media, probably due to increased awareness campaigns in the media in recent times. Other outlets such as the internet should be used in spreading information on female cancers especially in this time of smart phones. Similarly, in a study carried out in Saudi Arabia, results showed that 93.6% of participants had heard of breast cancer and a slightly lower proportion (30.4%) heard about it from mass media and more from the internet (40.4%) and Physicians (43.4%) [27]. Majority of the respondents also knew the most common symptom of breast cancer much better than the 36.6% reported in the Saudi study [27]. Almost one in 20 of our respondents did not know any symptoms thus revealing knowledge gaps. This shows there is a high awareness of breast cancer, but gaps in knowledge in terms of risk factors and symptoms of the disease. Regarding screening, most of the respondents were also aware of breast cancer screening, the purpose, and that it has benefits.

Majority of the respondents had also heard of cervical cancer, mainly from mass media showing the positive effect of the awareness campaigns in the media. But in Nigeria generally, many men and women do not know what cervical cancer is, nor where it occurs [28]. Likewise in a study done in Phalombe, Malawi majority had heard of cervical cancer in the study area [24] but in Kumasi, Ghana, a neighbouring Country in West Africa, most of the participants indicated that they had never heard of cervical cancer [20]. This disparity could be a result of the general lack of awareness about cancer in Ghana as that was a common reason that several participants gave for not being aware of cervical cancer. In addition, participants indicated that the discussion of an individual’s health problems was not common cultural behaviour [20].

Knowledge of the cause of cervical cancer was poor but better than reports from other African Countries, Kenya, (only 20.0%), Ghana, and Malawi where only one of the participants in each study was able to correctly identify HPV as the major risk factor for cervical cancer [20, 23, 24]. Our respondents were highly educated and so more likely to be exposed to such information. HPV is sexually transmitted and is a necessary cause of cervical cancer and so men should know this to understand their pivotal personal role in preventing the disease. Misconceptions about the cause of cervical cancer and risk factors such as too much sex, poor hygiene, and abortion among others were also common among our respondents as observed in other African countries [20, 24].

Poor knowledge of symptoms of cervical cancer was also reported in the Malawian study [24] and the major symptoms known to the respondents were similar to results from Uganda [29]. This shows that while some men were knowledgeable about some of the symptoms of cervical cancer, many were ignorant. The information spread by the various media should go beyond creating awareness but also give accurate information about common female cancers. With regards to cervical cancer screening, our respondents had better knowledge than their Ghanaian counterparts [17, 24].

The poor overall knowledge about breast and cervical cancers and screening seen in this study is consistent with the research findings in other African countries where only a few men were knowledgeable about female cancers [20, 30, 31]. Culturally, issues to do with reproductive health are regarded as feminine [32] and this invariably contributes to their poor knowledge.

Respondents in this study, just like in Malawi [24], were more open to their partners being screened for cervical cancer by a male health professional. This may be due to the highly educated status of our respondents in addition to the urban setting. Conversely, in the Ghanaian study, many of the participants who were married indicated that they would not be comfortable knowing that their wife was having a cervical cancer screening performed by a male doctor [20]. This is probably due to cultural reasons where another man should not see the nakedness of their wife let alone, examine a very private part such as the cervix. Some women, after cervical cancer screening, refuse treatment because they have to first consult their husbands [33], showing that the issue of cultural effects is also tolerated by womenfolk. These findings suggest that traditional systems have a role to play in improving male involvement.

Overall, the majority had a positive attitude towards breast and cervical cancer screening. However, while the men were involved with the general health of their partners, they were not commensurately involved in their screening for female cancers. To buttress their key role in decision-making, the majority testified that screening is a joint decision, yet, they had not done much in that regard. Most men had never provided for their partner to screen, they had not encouraged them nor accompanied them to screen for breast cancer or cervical cancers. This shows the unwillingness of the men to get involved in the reproductive health of their partners even though they want to be heavily involved in decision making. Women in Indonesia reported that their husbands had little or no involvement in screening for women’s cancers. Only 5 out of the 20 women interviewed testified that their husbands provided monetary support, accompaniment or encouraged them to screen. This qualitative study was however conducted only among Muslim women and covered both urban and rural areas [15].

In Ghana, several participants indicated that the cost of the screening test would be a major barrier to screening and only some would encourage their wives to go for screening [20]. In the study in Malawi, almost all the participants would encourage their spouses to be screened for cervical cancer. Most of them men insisted that they were responsible for granting permission for their wives to access screening services and some (up to one-fifth) even suggested male circumcision as a means of involvement while others mentioned accompaniment and encouragement [24]. This study was done in a rural setting where few of the men were educated up to secondary school level. Women have reported that intimate partner’s emotional and financial support, and social support increased screening for cervical cancer [34, 35]. In another rural qualitative study in Ghana, male partners provided financial, material, and emotional support to their wives during the screening and treatment of their partners’ cervical cancer while some abandoned them. Others expressed willingness to give support in the event of cancer diagnosis [17].

This inadequate male involvement is manifest in the poor female cancer screening practices of the women in LMICs [12]. Underutilization of cervical cancer screening services contributes to late diagnosis and treatment with consequent increased morbidity and mortality. The significant roles of older age and higher educational level of men in their involvement in female cancer screening are worthy of note. Cancer incidence is higher with older age of the woman and so the men were also conscious of that. Highly educated men are also more likely exposed to more information. Our results have also shown that male involvement had significant positive association with actual screening for breast and cervical cancers by their wives/partners. This is a very potent justification for involving men more in female cancer prevention in resource poor settings like ours. Planned interventions should therefore design appropriate messages, and also target younger and less educated men. Respondents’ knowledge about common female cancers did not influence their involvement but their attitude did, showing that having a good attitude to screening is important in improving involvement. Men need to change their mindset and embrace change in line with recent calls globally, for increased male involvement in reproductive health issues.

### Strengths and limitations of the study

This is one of the few studies that quantitatively examined male involvement in female cancer screening especially in the African region and results have shown that lack of spousal involvement likely constitutes a barrier to screening. There is also paucity of data from men as respondents and this study contributes to closing this gap. The community-based nature of the study where there is more diversity, is also a strength. Due to face-to-face interview, respondents may have over-reported positive responses which were not verified by their partners. The sample size may limit generalizability. Future studies incorporating qualitative aspects will help explore other factors affecting male involvement in female cancer screening.

## Conclusion

Male involvement in female partners’ screening for breast and cervical cancers was inadequate. Most of the respondents were also not knowledgeable about breast and cervical cancers, but they had a positive attitude towards screening. As part of attempts to improve male involvement in female partners’ screening to reduce the burden of breast and cervical cancers in women in Nigeria and similar settings, we recommend community-based programs designed for men, especially the younger and less educated, as well as strengthening mass media campaigns and using internet platforms to increase their participation in reducing the incidence of female cancers.

## Supporting information

S1 File (DOCX)

S1 Data (XLSX)
