# Supplementary material for: Male involvement in female partners’ screening for breast and cervical cancers in Southwest Nigeria
Source: PLoS One. 2023 May 10;18(5):e0284141. doi: 10.1371/journal.pone.0284141 (PMC10171596; doi:10.1371/journal.pone.0284141)
Supplement: S1 File — (DOCX) [file pone.0284141.s001.docx]

# QUESTIONNAIRE

**MALE INVOLVEMENT IN FEMALE PARTNERS’ SCREENING FOR BREAST AND CERVICAL CANCERS IN SOUTHWEST NIGERIA**

SECTION A: SOCIO- DEMOGRAPHIC & ECONOMIC CHARACTERISTICS

1. Age (as at last birthday) ……. years
2. Marital status
3. Single (B) Married/Co-habiting (C) Separated/Divorced/Widowed
4. How long have you been married/together? ........................................................
5. Highest level of education attained/completed
6. Non-formal (B) Primary school (C) Secondary school

(D) Post-secondary education

1. Ethnicity
2. Yoruba (B) Igbo (C)Hausa

(D) Others (please specify) ....................................................................

1. Religion
2. Christian (B) Muslim (C) Others (please specify) .........................
3. Employment status
4. Employed (B) Unemployed
5. Average monthly income/allowance (in Naira) ......................................................
6. Number of wives/female partners ...................................
7. Number of children fathered …….. children

SECTION B: INFORMATION ON WIFE/PARTNER

1. Age of your wife/partner ..…… years
2. Highest level of education attained by your wife/partner

(A) Non-formal (B) Primary school (C) Secondary school (D) Post-secondary education

1. Employment status of your wife/partner
2. Employed (B) Unemployed

SECTION C: HEALTH SEEKING BEHAVIOUR OF RESPONDENT

1. Have you ever gone for check-up in a health facility?

Yes No

1. Have you ever gone for cancer screening in a health facility? (e.g prostrate cancer)

Yes No

SECTION D: KNOWLEDGE OF BREAST CANCER

1. Have you ever heard of breast cancer?

Yes No

If No, skip to question 25

1. If yes, how did you hear about breast cancer? (first source of information)
2. Health professional (B) Friends/family (C)Traditional medicine
3. Mass Media (E) Survivors (F) Internet (G) Others
4. What do you understand by breast cancer?

A disease in which cells in the breast grow out of control

I don’t know

Others

1. What are the symptoms of breast cancer? (multiple answers allowed)

Change in size or shape of breast

Dimpling of breast

Nipple discharge

Change in areola

Breast or nipple pain

Lump in breast

Others

I don’t know

1. What are the risk factors for breast cancer? (multiple answers allowed)

Family history of breast cancer

Taking oral contraceptives (birth control pills)

Aging

Smoking and alcohol consumption

Genetics (genes)

Being obese or overweight after menopause

Early menstrual period (before age 12)

Lack of physical activity

Others

I don’t know

1. Is there any treatment for breast cancer?

Yes No

SECTION E: KNOWLEDGE OF BREAST CANCER SCREENING

1. Have you ever heard of breast cancer screening?

Yes No

If No, skip to question 25

1. What is the purpose of breast cancer screening?
2. Detection of lumps/tumors
3. I do not know
4. Others
5. Is there any benefit of screening regularly?

Yes No Not sure

SECTION F: KNOWLEDGE OF CERVICAL CANCER

1. Have you ever heard of cervical cancer before?

Yes No

If No, skip to question 34

1. If yes, how did you hear about cervical cancer? (first source of information)

(A) Health professional (B) Friends/family (C) Traditional medicine

1. Mass Media (E) Survivors (F) Internet (G) Others
2. What do you understand by cervical cancer?

Abnormal growth of cells in the cervix

I don’t know

Others

1. What is the cause of cervical cancer?

(A) Too much sex (B) Human papilloma virus (HPV) (C)Poor hygiene (D) Abortion (E) I don’t know (F) Others

1. What are symptoms of cervical cancer? (Multiple responses allowed)

Pelvic pain

Bleeding after intercourse

Bleeding after menstrual periods

Increased unusual vaginal discharge

Pain during sexual intercourse

I don’t know

Others

1. What are the risk factors for cervical cancer? (multiple responses allowed)

Human papiloma virus (HPV) infection

Having multiple sexual partners

Human immunodeficiency virus (HIV)

I do not know

Others

1. Is there any treatment for cervical cancer?

Yes No

SECTION G: KNOWLEDGE OF CERVICAL CANCER SCREENING

1. Have you ever heard of cervical cancer screening?

Yes No

If No, skip to question 34

1. What is the purpose of screening for cervical cancer?

Detection of abnormal changes to the cervix/early detection of precancerous cells

I do not know

Others

SECTION H: ATTITUDE TO SCREENING AND DISEASES OF BREAST AND CERVICAL CANCERS

For each statement below, indicate your level of agreement

|  | Strongly Agree | Slightly agree | Don’t agree at all |
| --- | --- | --- | --- |
| 1. Screening for breast cancer without first observing symptoms is a waste of time |  |  |  |
| 1. Screening for cervical cancer without observing symptoms is a waste of time |  |  |  |
| 1. Screening for breast cancer is necessary |  |  |  |
| 1. Screening for cervical cancer is necessary |  |  |  |
| 1. I don’t need to accompany my female partner to screen for breast cancer |  |  |  |
| 1. I don’t need to accompany my female partner to screen for cervical cancer |  |  |  |
| 1. I should not interfere with my partner’s screening for breast and cervical cancers |  |  |  |
| 1. I will not wait for long while accompanying my partner to screen for breast and cervical cancers |  |  |  |
| 1. I will never allow a male health professional screen my female partner for breast and cervical cancers |  |  |  |
| 1. My wife/partner does not need to obtain my approval before screening for breast and cervical cancers |  |  |  |
| 1. I will leave my wife/partner if she is ever diagnosed with breast cancer |  |  |  |
| 1. I will leave my wife/partner if she is ever diagnosed with cervical cancer |  |  |  |

SECTION I: MALE INVOLVEMENT IN FEMALE PARTNERS’ HEALTH

1. Have you ever accompanied your female partner to a health care facility? (for reasons such as she was ill, antenatal care, doctor’s appointment, etc)

Yes No

1. Have you ever stayed home to take care of your female partner when she was ill?

Yes No

1. Have you provided financially for your partner to visit a health specialist?

Yes No

SECTION J: MALE INVOLVEMENT IN SCREENING FOR BREAST AND CERVICAL CANCERS

1. Screening for breast and cervical cancers is the decision of:
2. The woman alone (B) The man alone (C) Both partners

(D) Others (please specify)..................................................................

1. Have you provided financially for your wife/partner to be screened for breast cancer?

Yes No

1. Have you provided financially for your wife/partner to be screened for cervical cancer?

Yes No

1. Have you ever sacrificed your time to accompany your wife/partner to be screened for breast cancer?

Yes No

1. Have you ever sacrificed your time to accompany your wife/partner to be screened for cervical cancer?

Yes No

1. Have you ever encouraged your wife/partner to screen for breast cancer?

Yes No

1. Have you ever encouraged your wife/partner to screen for cervical cancer?

Yes No

1. Has your wife/partner ever been screened for breast cancer?

Yes No I don’t know

If No, skip to question 58

1. If Yes, what was the result?

Positive Negative I don’t know

1. Has your wife/partner ever been screened for cervical cancer?

Yes No I don’t know

If No, skip to question 60

1. If Yes, what was the result?

Positive Negative I don’t know
